# The Effect of Prophylactic Intravenous Amiodarone Administration on Reperfusion Ventricular Fibrillation in Patients With Left Ventricular Hypertrophy Undergoing Cardiopulmonary Bypass Surgery: Protocol for a Randomized Double-Blind Clinical Trial

**DOI:** 10.2196/40115

**Published:** 2023-01-27

**Authors:** Xiaokai Zhou, Zhenfeng Zhang, Chanjuan Gong, Yin Fang

**Affiliations:** 1 Department of Anesthesia and Perioperative Medicine The First Affiliated Hospital of Nanjing Medical University Nanjing China

**Keywords:** amiodarone, cardiopulmonary bypass, left ventricular hypertrophy, ventricular fibrillation

## Abstract

**Background:**

Ventricular fibrillation (VF) is a common arrhythmia that occurs after the release of aortic cross-clamp (ACC) in patients undergoing cardiopulmonary bypass (CPB) surgery. Repeated defibrillation and long duration of VF could increase myocardial injuries. In patients with left ventricular hypertrophy (LVH), VF is easier to occur and more difficult to be terminated. Amiodarone, known as class III antiarrhythmic agent, has the prominent properties of converting VF and restoring the sinus rhythm. Before ACC release, administration of amiodarone has been confirmed useful to reduce occurrence of VF. However, few studies are focused on the effect of amiodarone before ACC release on reducing VF in patients with LVH.

**Objective:**

This study aimed at determining the efficacy of prophylactic intravenous amiodarone administration on reperfusion VF after release of ACC in patients with LVH undergoing CPB surgery.

**Methods:**

This will be a prospective, randomized, double-blind, placebo-controlled trial. The trial will enroll 54 patients with LVH aged 18-75 years who will undergo CPB surgery. All eligible participants will be randomly allocated to either the amiodarone or placebo group by using the block randomization in a 1:1 ratio. The primary end point will be the incidence rate of VF 30 minutes after ACC release and be assessed using the Fisher exact test. All data will be analyzed in accordance with the intention-to-treat principle.

**Results:**

The study began in August 2022, and the data collection will take place for the next 2 academic years. As of this writing, 21 participants have already been recruited for the study.

**Conclusions:**

With this trial, we are hoping to demonstrate that prophylactic infusion of amiodarone before ACC release could reduce the occurrence of reperfusion VF in patients with LVH.

**Trial Registration:**

ClinicalTrials.gov ChiCTR2000035057; https://www.chictr.org.cn/showprojen.aspx?proj=57145

**International Registered Report Identifier (IRRID):**

DERR1-10.2196/40115

## Introduction

### Background

Ventricular fibrillation (VF) is commonly seen after aortic cross-clamp (ACC) release during cardiopulmonary bypass (CPB) surgery [1]. It has been reported that the incidence rate of VF after ACC release ranges from 45% to 100% [1-6]. Left ventricular hypertrophy (LVH) has been found to increase the incidence rate of VF after ACC release, which is more difficult to be terminated [7]. Internal electrical defibrillation is an effective method to eliminate VF but repeated shock and long duration of VF could aggravate myocardial injury and reduce the patient’s prognosis [8].

There are different factors leading to VF after ACC release including surgical trauma, insufficient myocardial protection, inadequate intracardiac deairing or left ventricular vent, abnormal electrolyte and acid-base metabolism, delayed rewarming before ACC release and myocardial reperfusion injury after ACC release, etc. Among these, myocardial reperfusion injury is the main reason resulting in VF after ACC release [9]. The ischemia of cardiomyocytes could cause the increase in extracellular potassium ions. When the blood supply is restored, the uneven recovery of extracellular potassium ions makes the cell membrane potentially unstable, which would easily lead to increased self-discipline or triggered arrhythmias [10]. At the same time, myocardial ischemia-reperfusion can increase intracellular calcium levels, resulting in delayed depolarization of cardiomyocytes and triggered electrical activity, which also induces ventricular arrhythmias. Furthermore, α-adrenergic receptors are activated in reperfusion cardiomyocytes over a short period of time, contributing to increased myocardial autonomic activity and intracellular calcium levels [11]. The ischemic cardiomyocytes gradually and slowly recover their activities after the blood resupply, during which they are prone to reentrant arrhythmias, and the effective refractory period of cardiomyocytes in ischemic regions decreases, which also provides conditions for the occurrence of reentrant arrhythmias [12]. Therefore, both reentrant and nonreentrant arrhythmias play a role in the occurrence of ischemia-reperfusion VF.

Long duration of VF has many side effects on cardiomyocytes after ACC release. A study showed that coronary artery blood supply was redistributed during VF, which resulted in subendocardial myocardial ischemia and cardiac dysfunction [13]. With the prolongation of VF duration, the resistance of subendocardial vessels increased gradually, which worsened the subendocardial ischemia of cardiomyocytes. Besides, myocardial edema also developed with the long duration of VF [14]. The ventricular wall tension and venous pressure would increase during VF and higher perfusion pressure would be required to achieve normal blood supply in CPB. Frequent and irregular myocardial contractions and increased pressure in the cardiac cavity could result in a higher oxygen consumption of myocardium. The abovementioned factors lead to the imbalance of oxygen supply and demand in myocardium, which seriously destroy the function of cardiomyocytes. Moreover, Khuri et al [15] discovered that metabolic acidosis was more severe in cardiomyocytes during VF and the postoperative mortality rate was higher in a group of patients. Creatine kinase–myocardial band (CK-MB) is a specific marker of myocardial injury. After ACC release, the postoperative CK-MB of patients with VF was significantly higher than that of patients without VF, and the degree of elevation was 75% related to the duration of VF [16].

Defibrillation could also damage the myocardium. Doherty et al [17] found that when the energy of defibrillation was more than 30 J, the damage of cardiomyocytes increased and the blood flow in cardiomyocytes decreased. Multiple defibrillations significantly increased myocardial damage compared with single defibrillation. The shorter time interval of each defibrillation also resulted in greater myocardial injuries. It was observed under electron microscope that the gaps between myocardial intercalated discs increased after defibrillation, thus affecting the myocardial contractile function. A clinical study showed that myocardial injury markers, cardiac troponin T, cardiac troponin I, and CK-MB were significantly elevated after cardiac shock, which further confirmed the myocardial injury owing to internal electrical defibrillation [18].

In patients with LVH, the volume of cardiomyocytes expands, but the corresponding coronary arteries do not increase, resulting in absence of subendocardial capillaries. The end-diastolic pressure and systolic cardiac function also increase, leading to increased myocardial oxygen consumption. These histological changes give rise to the imbalance of myocardial oxygen supply and demand, and ischemia of subendocardial myocardium, which reduces the tolerance of myocardium to ischemia-reperfusion [19]. Owing to the special structure of LVH, the perfusion fluid of CPB could not completely reach the subendocardial cardiomyocytes. Compared with the normal heart, the effect of myocardial protection during CPB is usually inadequate. The incidence of VF after ACC release increased significantly, and the energy and frequency of cardiac shocks in hypertrophic myocardium increased as well [20]. Thus, reducing the occurrence of VF has an important clinical significance.

Amiodarone is a class III antiarrhythmic agent with multiple electrophysiological effects, which has been used clinically in the management of both atrial and ventricular arrhythmias. Through hepatic metabolism, amiodarone is transformed to desethylamiodarone, which has the property of antiarrhythmic effects. Amiodarone is highly lipid soluble and is widely stored in fat, muscle, skin, liver, and lungs with a very long elimination half-life, averaging about 58 days [21]. The predominant antiarrhythmic effect of amiodarone is the inhibition of IKr and IKs channels leading to a prolongation of myocardial repolarization homogeneously. Amiodarone also slows heart rate and atrioventricular nodal conduction through inhibiting calcium channels and β-receptors. In addition, it also prolongs refractoriness and reduces intracardiac conduction velocity by blocking sodium channel [22,23]. It has been confirmed that intravenous amiodarone could protect myocardium from ischemia-reperfusion and prevent VF caused by reperfusion [24-26]. During ischemia-reperfusion, amiodarone can improve the metabolic efficiency of cardiomyocytes and reduce the repolarization dispersion of cell membrane, which is one of the main factors causing ventricular arrhythmias.

Previous studies have shown the effectiveness of amiodarone on reducing the incidence rate of VF after ACC release [2,3,27,28]. Samantaray et al [3] reported that during coronary artery bypass grafting surgery, 150 mg of amiodarone was given 3 minutes before aortic opening, which decreased the incidence of VF after ACC release compared with the placebo-controlled group. However, in the study of Ayoub and colleagues [2], the administration of amiodarone 2 minutes before aortic opening failed to reduce the incidence of VF after ACC release during coronary artery bypass grafting surgery, but lidocaine instead reduced the occurrence of VF. Mauerman et al [27] also found that neither 300 mg of amiodarone nor 1.5 mg/kg of lidocaine reduced the occurrence of VF. Furthermore, the study of Yilmaz et al [28] showed that both 300 mg of amiodarone and 1.5 mg/kg of lidocaine decreased VF after ACC release compared with the placebo-controlled group, but there was no significant difference in reducing VF between amiodarone and lidocaine groups. Although the results from the abovementioned clinical studies are controversial, they generally hint that amiodarone has a therapeutic effect on reperfusion VF after ACC release.

In a case report of Morita et al [7], a 66-year-old female patient underwent aortic valve replacement due to severe aortic stenosis, and preoperative transthoracic echocardiogram showed the thickness of ventricular septal and left ventricular posterior wall up to 18 mm. Refractory VF after ACC release occurred and lidocaine infusion and multiple cardiac shocks were both ineffective. Thereafter, 150 mg of amiodarone was given through the root of aorta, and finally VF was terminated by one defibrillation. Suzuki et al [20] also reported a similar case of using amiodarone in terminating reperfusion VF during cardiac surgery. There is only a clinical trial about amiodarone in reducing reperfusion VF in patients with LVH [29]. In this trial, patients with LVH undergoing aortic valve replacement were randomly assigned to receive a bolus of 150 mg of amiodarone plus maintenance dose of 30 mg/hour or a bolus of 1 mg/kg of lidocaine plus maintenance dose of 1 mg/kg/hour after anesthetic induction. The incidence rate of VF was significantly lower in the amiodarone group than in the lidocaine group (20.6%, 7/34 vs 50%, 17/34, *P*=.02). Meanwhile, amiodarone could reduce the level of interleukin-6 and tumor necrosis factor after operation. In addition to the abovementioned studies, no more studies were focused on this area.

### Objective

The objective of this study is to explore the outcomes of prophylactic intravenous amiodarone administration on reperfusion VF after ACC release in patients with LVH undergoing CPB surgery.

This study also concentrates on the effect of amiodarone on hemodynamic state after weaning from CPB in patients with LVH, which few previous studies have examined.

## Methods

### Study Design and Setting

**Figure 1:**
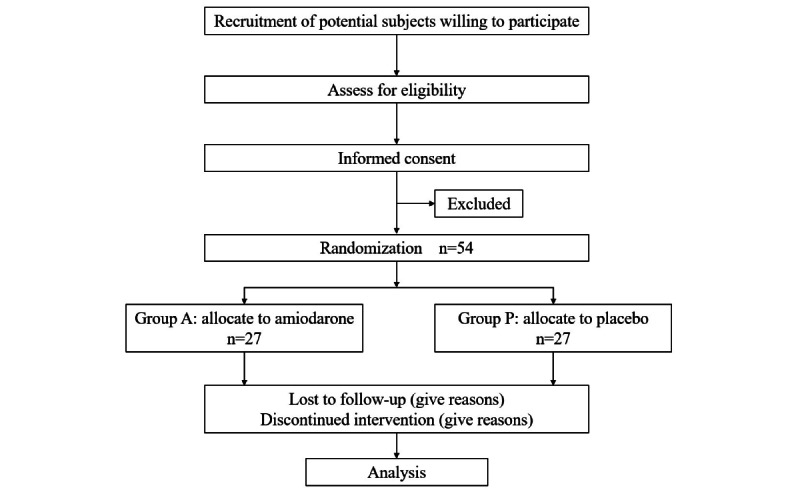
Trial flow chart.

This study is a prospective, single-center, randomized, double-blinded, placebo-controlled clinical trial to investigate effect of perioperative intravenous amiodarone on reperfusion VF after release of ACC in patients with LVH undergoing CPB. The study will be conducted in the First Affiliated Hospital of Nanjing Medical University, Nanjing, China. A schematic of the trial protocol is presented in Figure 1.

### Study Population

Fifty-four patients who meet the eligibility criteria will be recruited and randomly divided into 2 groups, including amiodarone group (group A) and placebo-controlled group (group P), 27 cases in each group. Informed consent will be obtained from all participants (Textbox 1).

**Inclusion and exclusion criteria of participants.**

**Inclusion criteria**

Patients included in the trial are as follows:

- Subjects undergoing cardiopulmonary bypass surgery
- Aged between 18 and 75 years
- With left ventricular hypertrophy diagnosed by transthoracic echocardiogram preoperatively
- American Society of Anesthesiologists class I-III

**Exclusion criteria**

Patients fulfilling one or more of the following criteria will not be included:

- Preoperative use of class I or III antiarrhythmic drugs
- Corrected QT interval of ≥480 milliseconds or QT of ≥500 milliseconds
- Left ventricular ejection fraction <30%
- With thyroid dysfunction
- With electrolyte imbalances
- With hepatic or renal dysfunction
- With pulmonary interstitial fibrosis
- Allergic to amiodarone
- Undergoing emergency surgery
- Undergoing redo surgery

### Randomization and Blinding

Thirty minutes before ACC release, the enrolled participants who meet the eligibility criteria will be randomly allocated in a 1:1 ratio to 2 groups, namely, group A (treated with amiodarone) and group P (placebo control). Block randomization will be used with a block size of 4, which will be implemented with SPSS (version 22.0; IBM Corp) by an independent statistician, and the randomization numbers will be given to an external data manager with no involvement in the study procedures and concealed on password-protected computer.

When a patient meets the eligibility criteria, the external data manager will return the corresponding allocation to the nurse anesthetist. The drugs will be prepared by the nurse anesthetists according to the allocation information, packaged with the same looking and labeled with numbers, and injected by the anesthesiologists. Both anesthesiologists and patients will be blinded to the regimen. The anesthesiologist will be notified of the study group by the nurse anesthetists in case of emergency. However, the nurse anesthetists have no role in assessing the treatment actions, analyzing, or interpreting the data.

### Interventions

#### Treatment Regimen of Amiodarone

Thirty minutes before ACC release, the trial drugs will be administered through the central venous line. In group A, 150 mg of amiodarone will be diluted to 20 mL normal saline and pumped in 5 minutes. In group P, 20 mL normal saline will be pumped in 5 minutes (Table 1).

**Table 1:** Schedule for enrolment and intervention per cluster.

Time point^a^		Enrollment	Allocation	Close-out			
		–*t*_1_	0	*t*_1_	*t*_2_	*t*_3_	*t*_4_
**Enrollment**							
	Eligibility screen	✓^b^					
	Informed consent	✓					
	Allocation		✓				
**Interventions**							
	Group A^c^			✓			
	Group P^d^			✓			
**Assessments**							
	Baseline demographic information	✓					
	Primary end point^e^				✓		
	Secondary end point^f^				✓	✓	✓

^a^Time point: *t*_1_: baseline; *t*_1_: intervention period, 30 minutes before aortic cross-clamp (ACC) release; *t*_2_: 10 minutes after ACC release; *t*_3_: 15 minutes after weaning from cardiopulmonary bypass; *t*_4_: the end of surgery.

^b^✓: refers to crucial events at corresponding points in time.

^c^Group A: amiodarone group.

^d^Group P: placebo-controlled group.

^e^Primary end point: occurrence of ventricular fibrillation (VF).

^f^Secondary end point: *t*_2_: the number and maximum energy of defibrillations to terminate VF; *t*_3_: heart rate, mean arterial pressure, mean pulmonary arterial pressure, central venous pressure; *t*_4_: the total dosages of vasoactive agents.

#### Anesthesia Protocols

All the patients will fast for 8 hours and abstain from water for 4 hours preoperatively and receive standardized general anesthesia. In the operating room, the patients will be routinely monitored including 5-lead electrocardiograph, peripheral blood oxygen saturation, invasive arterial blood pressure, bispectral index (BIS), and end tidal CO_2_, which will be recorded every 3 minutes in each patient’s electronic medical record. The induction of anesthesia will be performed as follows: 0.05 mg/kg midazolam, 0.3 mg/kg etomidate, 0.15 mg/kg cis-atracurium, and 4-6 μg/kg fentanyl. Approximately 5 minutes after induction, patients will be intubated and ventilated to maintain the end tidal CO_2_ at 35-45 mm Hg. Then, the ultrasound-guided central vein catheterization will be conducted via the right internal jugular or subclavian vein. Swan-Gans catheter will be floated to pulmonary artery through the right internal jugular. Thereafter, transesophageal echocardiogram will be inserted for the intraoperative cardiac estimations. The patients continued receiving anesthesia by inhalation of sevoflurane 1%-2% and continuous pump of propofol 1-2 mg/kg/hour, dexmedetomidine 0.5 μg/kg/minute, and cis-atracurium 0.05-0.1 mg/kg/hour. The total dosage of fentanyl will be 30-50 μg/kg. During continuation of anesthesia, the BIS will be maintained between 40 and 60. Inotropic agents, vasopressors, vasodilators, or fluid administration will be applied to achieve the following hemodynamic status: a mean blood pressure of 50-80 mm Hg, cardiac index (CI) ≥2.2 L/min/m^2^, and urine output ≥1.0 mL/kg/hour.

#### Surgical and CPB Procedures

The surgical procedures will be performed via median sternotomy with heparinization and standard CPB. The cannulation sites for CPB are at the discretion of the cardiac surgeon and dependent on the operation. The aortic cannulation is obtained with a patient size–appropriate cannula. Venous cannulation is obtained with a single 2-stage cannula in the right atrium or separate cannula in the superior and inferior vena cava. Anticoagulation is started with 400 μg/kg of heparin and maintained according to an activated clotting time of >480 seconds. CPB is initiated using a membrane oxygenator and the circuit is primed with 1500 mL of acetate Ringer solution and 200 mL of 50% human serum albumin. Patients are perfused by a nonpulsatile roller pump at a flow rate of 2.0-2.4 L/minute/m^2^ and with mean arterial pressure (MAP) of 50-80 mm Hg. Moderate systemic hypothermia around 30-32 °C is maintained during CPB. Myocardial protection is achieved using intermittent antegrade or retrograde infusions of histidine-tryptophane-ketogluterate cardioplegia. Cardioplegic infusion is repeated every 120 minutes or when electrical activity of the heart is detected. After weaning from CPB, reversal of heparin is achieved with protamine.

#### Management of VF

Thirty seconds before ACC release, 1 mg/kg of esmolol will be given to all patients. When VF occurs after ACC release, internal electrical defibrillation with 20 J will be performed in the first attempt. If not successful, the second defibrillation with 20 J will be attempted after reusing 0.5-1 mg/kg of esmolol. If VF persists, the third defibrillation with 30 J will be performed after giving 1 mg/kg of lidocaine. If VF termination is not achieved, the fourth defibrillation with 40 J will be attempted after administration of 50 mg of amiodarone. If VF still exists, administration of 100 mg of amiodarone and defibrillation with 50 J will be repeated until VF terminates.

### End Points

The primary end point is the incidence rate of VF 10 minutes after ACC release. The secondary end points include the duration of VF, the number and maximum energy of defibrillations needed to terminate VF, the use of other antiarrhythmic agents, and epicardial temporary pacemakers after ACC release. Besides, the secondary end points also include heart rate, MAP, mean pulmonary arterial pressure, central venous pressure and CI 15 minutes after anesthetic induction and weaning from CPB, and the speed of vasoactive agents at the end of CPB and surgery and their total dosages.

### Sample Size Estimation

The sample size is calculated based on the primary end point: the incidence rate of VF 10 minutes after ACC release using PASS (version 15.0; NCSS). According to the published studies, the incidence rate of VF after ACC release ranges from 45% to 100% [1-6]. We assumed that the incidence rate of VF 10 minutes after ACC release would be 70% in the placebo-controlled group and amiodarone could decrease the rate to 30%. To reach statistical significance with a power of 80% and an α value of .05 (2-sided), 21 patients are assessed to be needed in each group. Assuming a 20% dropout or missing data rate, we will recruit 27 patients in each group for a total of 54 patients.

### Statistical Analysis

All data will be analyzed using SPSS (version 22.0; IBM Corp) and in accordance with the intention-to-treat principle, beginning immediately after randomization. All statistical tests will be considered significant if *P*<.05. Only 2-sided tests will be used. Baseline characteristics will be described and compared by using the Fisher exact test for the categorical variables and 2-sample *t* test or Wilcoxon rank sum test depending on the normality of the data distribution for the continuous variables. The continuous variables will be described by mean and SD values if normally distributed or median and interquartile range if not. The normality of distributions will be checked graphically and using the Kolmogorov-Smirnov test. If there is a violation of distribution assumption, appropriate transformation will be used. The categorical variables will be expressed as frequencies and percentages.

For the primary end point, the incidence rate of VF 10 minutes after ACC release will be compared by using the chi-square test. For the secondary end points, heart rate, MAP, mean pulmonary arterial pressure, central venous pressure, and CI will be compared between 2 groups by using a repeated measures ANOVA model during the 2 observation times. Other continuous secondary end point variables will be compared between 2 groups by using a 2-sample *t* test or a Wilcoxon rank sum test depending on the normality of the data distribution. The chi-square test or Fisher exact test will be used to compare categorical secondary end point variables.

If data are missing at random, the analyses will be carried out using multiple imputations. The multiple imputation procedure will be based on all available data for that patient and be conducted using the chained equation approach. If data are not missing at random, the analyses will be conducted using available data with appropriate interpretational reservations. We will also evaluate the data using the intention-to-treat principle and per-protocol data set and compare the analysis results to estimate reliability of our analytical results.

### Adverse Events Observation

During trial agent infusion, if the mean arterial pressure is less than 50 mm Hg, 20-40 μg of norepinephrine will be administered through the CPB venous reservoir. After recovery of the autonomous cardiac rhythm, if the heart rate is <60 bpm or a type II second-degree or third-degree atrioventricular block develops, 0.5-1 mg of atropine will be used intravenously, and if it does not work, epicardial temporary pacing will be performed. If the QT interval is >440 milliseconds or the corrected QT interval is >400 milliseconds, 0.5-1 mg of atropine will be given to increase heart rate to >70 bpm and blood potassium will be ensured at >4.0 mmol/L.

### Data Collection and Management

The data will be collected from paper case report forms (manually counter checked with source files by the data entry personnel) by the investigator and given to the statistician to analyze the data. The data will be recorded by their specific ID number instead of participant’s name throughout the study unless otherwise specified. Access to data will be restricted to the investigators who signed the confidential disclosure agreement or to the institutional or governmental auditors during the study. All original documents and files will be archived for at least 5 years to allow inspection after the trial has ended. The process will be monitored by the Institutional Ethics Committee of the First Affiliated Hospital of Nanjing Medical University.

### Ethical Considerations

This study has been approved by the Institutional Ethics Committee of the First Affiliated Hospital of Nanjing Medical University (2020-SR-175) and registered with the Chinese Clinical Trial Registry (ChiCTR2000035057). Informed consent will be obtained from all participants before recruiting to the study.

## Results

The study began in August 2022, and the data collection will take place for the next 2 academic years. To obtain the total study sample, a total of 54 participants will be recruited. At present, 21 participants have already been recruited for the study.

## Discussion

The clinical trial is designed in order to investigate the efficacy of prophylactic intravenous amiodarone administration on reperfusion VF after release of ACC in patients with LVH undergoing CPB surgery.

Reperfusion VF is a common arrhythmia encountered after ACC release in patients undergoing CPB surgery. Repeated defibrillation and long duration of VF could increase myocardial oxygen consumption, damage cardiomyocytes, and decrease the patients’ prognosis postoperatively. In patients with LVH, cardiac protection is often not as effective as the normal heart during CPB, and thus reperfusion VF is easier to occur and more difficult to be terminated.

Amiodarone, known as class III antiarrhythmic agent, has the properties of converting VF and restoring the sinus rhythm and can also be administered in patients with structural heart diseases. Before ACC release, administration of amiodarone has been confirmed useful to reduce occurrence of reperfusion VF. However, few proofs supported the effect of amiodarone before ACC release on reducing VF in patients with LVH. With this trial, we are hoping to further demonstrate that prophylactic infusion of amiodarone could reduce the occurrence of reperfusion VF in patients with LVH.

The limitation of this study may include the following aspects. First, this is a single-center trial with Asian patients in which there might be a selection bias. Second, the sample size is relatively small. However, according to the clinical experience in our center, the incidence of reperfusion VF is similar to those documented in the literature. So, the sample size could prove our hypothesis. We hope that in the future, a multicenter clinical trial with larger sample size could be performed to further elucidate this problem.

This study is believed to provide more proof regarding how to avoid reperfusion VF in patients with LVH undergoing CPB surgery, reducing myocardial damages occurred in those patients.

## Abbreviations

ACC: aortic cross-clamp
BIS: bispectral index
CI: cardiac index
CK-MB: creatine kinase-myocardial band
CPB: cardiopulmonary bypass
LVH: left ventricular hypertrophy
MAP: mean arterial pressure
VF: ventricular fibrillation

## Data Availability

Access to data will be restricted to the investigators who signed the confidential disclosure agreement or to the institutional or governmental auditors during the study. All original documents and files will be archived for at least 5 years to allow inspection after the trial has ended.
